# Assessing fidelity of a community based psychosocial intervention for people with mild dementia within a large randomised controlled trial

**DOI:** 10.1186/s12877-021-02070-8

**Published:** 2021-02-11

**Authors:** Kirsty Sprange, Jules Beresford-Dent, Gail Mountain, Claire Craig, Clare Mason, Katherine Berry, Jessica Wright, Shazmin Majid, Ben Thomas, Cindy L. Cooper

**Affiliations:** 1 Nottingham Clinical Trials Unit, Faculty of Medicine, University of Nottingham, Building 42, Nottingham, NG7 2RD UK; 2 University of Bradford, Bradford, West Yorkshire BD7 1DP UK; 3 Sheffield Hallam University, City Campus, Sheffield, S1 1WB UK; 4 Manchester Academic Health Science Centre, The University of Manchester, Oxford Rd, Manchester, M13 9PL UK; 5 Sheffield Clinical Trials Research Unit, School of Health and Related Research, University of Sheffield, Sheffield, S1 4DP UK; 6 Institute of Mental Health, Jubilee Campus, University of Nottingham Innovation Park, Triumph Road, Nottingham, NG7 2TU UK

**Keywords:** Complex intervention, Self-management, Dementia, Fidelity assessment

## Abstract

**Background:**

Understanding intervention delivery as intended, particularly in complex interventions, should be underpinned by good quality fidelity assessment. We present the findings from a fidelity assessment embedded as part of a trial of a complex community-based psychosocial intervention, Journeying through Dementia (JtD). The intervention was designed to equip individuals with the knowledge and skills to successfully self-manage, maintain independence, and live well with dementia and involves both group and individual sessions. The methodological challenges of developing a conceptual framework for fidelity assessment and creating and applying purposely designed measures derived from this framework are discussed to inform future studies.

**Methods:**

A conceptual fidelity framework was created out of core components of the intervention (including the intervention manual and training for delivery), associated trial protocols and pre-defined fidelity standards and criteria against which intervention delivery and receipt could be measured. Fidelity data collection tools were designed and piloted for reliability and usability. Data collection in four selected sites (fidelity sites) was via non-participatory observations of the group aspect of the intervention, attendance registers and interventionist (facilitator and supervisor) self-report.

**Results:**

Interventionists from all four fidelity sites attended intervention training. The majority of group participants at the four sites (71%) received the therapeutic dose of 10 out of 16 sessions. Weekly group meeting attendance (including at ‘out of venue’ sessions) was excellent at 80%. Additionally, all but one individual session was attended by the participants who completed the intervention. It proved feasible to create tools derived from the fidelity framework to assess in-venue group aspects of this complex intervention. Results of fidelity assessment of the observed groups were good with substantial inter-rater reliability between researchers KAPPA 0.68 95% CI (0.58–0.78). Self-report by interventionists concurred with researcher assessments.

**Conclusions:**

There was good fidelity to training and delivery of the group aspect of the intervention at four sites. However, the methodological challenges of assessing all aspects of this complex intervention could not be overcome due to practicalities, assessment methods and ethical considerations. Questions remain regarding how we can assess fidelity in community-based complex interventions without impacting upon intervention or trial delivery.

**Trial registration:**

ISRCTN17993825.

**Supplementary Information:**

The online version contains supplementary material available at 10.1186/s12877-021-02070-8.

## Background

Despite growing recognition of the value of psychosocial interventions to assist people to adapt and live well with dementia [1, 2] there is still a paucity of high quality research evidence for intervention effectiveness [3]. These psychosocial interventions are by their nature complex and therefore present evaluation challenges including the extent to which the intervention is delivered as intended. Factors to take into account include the impact of context upon intervention delivery [4] and whether the desired behaviour change is achieved [5]. Interest in intervention fidelity originated in response to treatment integrity concerns and demands for accountability in research. This was followed by a wider focus upon compliance (the extent to which those taking part in a trial follow the protocol) and increase in use of strategies such as intervention manualisation and training for systematic implementation and maintenance of fidelity [6, 7]. Reporting fidelity is now considered essential to determine the credibility, validity, and replicability of findings [8, 9]. As part of well-designed randomised controlled trials, fidelity studies can also help to establish intervention effectiveness and thereby support implementation into practice [10]. Embedded fidelity studies are therefore a feature of recent psychosocial dementia trials [11, 12]. Mixed-method trial designs are recommended to capture intervention effectiveness as well as fidelity of delivery [13, 14].

Intervention manualisation as well as intervention specific training and supervision can improve compliance and outcomes [15], thereby enhancing fidelity [6, 16]. Researchers are also encouraged to adopt outcome measures that offer validity [7, 17] and that can be measured consistently through rigorous processes such as randomised controlled trials to evidence effectiveness. However, the nature of complex tailored interventions could be considered in opposition to the idea of measuring consistent delivery [18]. For example, complex interventions often rely upon the judgements of those delivering the intervention to make any necessary adaptations. Therefore, fidelity assessment seeks to understand to what extent adaptations can be made without the intervention becoming different from what was intended [19]. Creation and application of appropriate measures to assess adaptation can improve our understanding of how different intervention components impact upon delivery and receipt of the intervention in context [20]. However, the uniqueness of complex interventions usually demands the creation of purposely designed measures for fidelity assessment, which lack demonstrable psychometric properties. Consequently bespoke fidelity measures and evaluation criteria (behaviours and activities observed) need to be formulated and located in the theoretical underpinnings, as well as in the aims and core content of the specific intervention [4].

This paper reports the results of an embedded fidelity assessment within a large randomised controlled trial to explore delivery of a psychosocial intervention, Journeying through Dementia [21]. The primary aim of the fidelity assessment was to evaluate how well the Journeying through Dementia intervention was delivered according to the trial protocol and intervention manual applying pre-defined fidelity standards. To achieve this, it was necessary to create appropriate fidelity documents and materials. This multi-component community-based intervention was co-designed by people living with dementia [22]. It includes mechanisms to increase independence, self-efficacy and effective problem solving in people living with early-stage dementia, thereby enabling individuals to live as well as possible with the condition. This paper provides a working example of how fidelity assessment using a range of methods can be successfully implemented using purposely designed measures within the context of a trial of a complex psychosocial intervention, Journeying through Dementia, to inform future studies.

## Methods

### Trial design

We conducted a pragmatic, two-arm parallel group, randomised controlled trial of the Journeying through Dementia intervention alongside usual care or usual care alone which included an embedded fidelity assessment and qualitative sub-study [21]. Patients were randomised using a secure, centralized, internet-based interface. The assignment sequence was computer-generated with block size of 4 in a 1:1 ratio and stratification according to trial site. The primary outcome for the effectiveness study was quality of life, measured by the Dementia Related Quality of Life (DEMQOL) at 8 months post-randomisation. A range of secondary outcomes measured other key components of the intervention. These were health and social care resource use; self-efficacy; well-being; self-management; activities of daily living and quality of life. A total of 480 participants with mild dementia (score of > 18 on MMSE) were recruited and randomised of which 241 were randomised to receive the intervention. The Trial was registered ISRCTN17993825, Date registered 11 Oct 2016.

### Sample

A convenience sample of four out of the 13 recruiting sites participating in the trial were approached and consented to take part in the fidelity assessment. Site selection for fidelity sites was pragmatic with criteria being that sites were actively recruiting and delivering the intervention. Geographical location and population size were also considered to ensure that selected sites were representative of those that took part in the trial. Unless otherwise stated data reported was collected during a 12-week programme at each of the four participating sites.

### Intervention

The Journeying through Dementia intervention, underpinned by social cognitive theory [23], is a manualised self-management multi-component intervention designed in consultation with people with dementia [22, 24]. It consists of 12 consecutive facilitated weekly group meetings held in a regular venue (in-venue) and four individual sessions with a facilitator to focus on personal goals. A minimum of three of the 12 group meetings are activities held in the community outside the regular venue (out of venue) to consolidate learning and practice neglected skills with support from others. Participation is designed to promote independence and self-management and support meaningful occupation including social interaction. Although manualised, the intervention enables tailoring of activities according to the needs of individuals and the group. This is achieved through a layered approach to learning and behaviour change within each of the different components (in-venue group meetings, individual sessions and out of venue activities). The intervention aims to elicit behaviour change through supporting greater self-efficacy, increased self-management and effective problem solving [25].

Mitchie’s theory of behaviour change informed the intervention design and delivery [26]. Within this theoretical framework, the anticipated change was improved self-management and engagement in meaningful activity. The theory emphasizes the importance of capability addressed by imparting knowledge and training. Motivation is addressed by increasing understanding through experiential learning which leads to behaviour change being associated with positive feelings. Opportunity for change is addressed by enabling participants to experience change within the groups as well as in the community.

Prior to the trial starting it was agreed a therapeutic dose would constitute 10 sessions attended out of the potential 16 (group and individual sessions). This was a pragmatic decision taken by the trial team with clinical advice which took account of experience of delivering similar interventions.

#### Training

An important aspect of fidelity is to reduce variability between interventionists. Inadequate or limited training can be factors in poor fidelity [7, 8]. Therefore, to maximize intervention delivery as intended a two-day training package was prepared for the purposes of the trial. It was delivered by the manual author, a highly experienced trainer with support from a second tutor with experience working with people with dementia. A standardised approach to training promoted consistent future delivery by interventionists. Following recommendations and learning from other studies [24, 27], training included experiential work to practice and model the intervention. The two-day training was intended to be delivered as close as possible to the beginning of intervention delivery for those who attended.

#### Supervision

Once intervention delivery commenced, all facilitators received weekly supervision from an experienced clinical professional from within their organisation but whom did not have experience of the intervention. This was not ideal as ‘real world’ delivery supervisors would have direct experience of delivering the intervention. To account for this, members of the trial team, experienced in delivering and supervising psychosocial interventions within trials provided supervision to site based supervisors via monthly face-to-face or phone meetings [28]. Those in the site supervisor role were encouraged to attend day one of the two-day training alongside facilitators from their site. This was to ensure that all interventionists received the same information about the intervention, and to build relationships and support shared learning. In addition, all supervisors attended a separate half-day training session led by the trial team to discuss their role and raise any concerns. A supervision protocol (see supplementary document 1) was provided to all sites for the purposes of the study and to support intervention fidelity.

### Aims and objectives of the fidelity assessment

The aim of the fidelity assessment was to evaluate how well the Journeying through Dementia intervention was delivered according to the trial protocol and intervention manual, applying pre-defined fidelity standards and criteria against which to measure these standards. The conceptual framework for this assessment is presented in Table 1. This framework was purposely designed using quality assurance parameters for provider training, delivery, receipt, and enactment based on criteria identified by the Behaviour Change Consortium [8] and NICE guidance on behaviour change [29].

**Table 1:** Fidelity conceptual assessment strategy for Journeying through Dementia

Parameter	Standard	Fidelity criteria	Measure of criteria/assessment tool
**Facilitator training**	Standardised training	All facilitators and supervisors receive the same training delivered in a similar way.	• Two-day training delivered by the same trainer(s) • Attendance registers for Two-day training • Researcher Two-day training checklist • Trainee Two-day training checklist
	Facilitator skill acquisition	All facilitators understand and engage with the intervention training in a similar way.	• Completion of training exercises by facilitators • Researcher training checklist • Trainee training checklist
**Intervention delivery**	Standardised delivery	All facilitators using the same intervention content: • The manual • Individual sessions • Goal setting • Out of venue activities	• Use of manualised intervention (Observations) • Facilitator – Group meeting checklist • Facilitator – Individual session checklist • Researcher - Group meeting checklist
	Minimise drift in skills/ delivery	Compliance to training content and delivery within and across sites over time.	• Researcher - Group meeting checklist • Record of provision of supervision • Supervision checklist (Supervisors and Facilitators) • Research supervision record
**Receipt of intervention**	Participant attendance and engagement	Numbers of participants attending the intervention. All participants taking part in the group meetings and activities.	• Attendance at group in-venue sessions (Register) • Attendance at Individual sessions (Register) • Use of manualised intervention (Observations)
	Comparable treatment	All participants receive the same intervention tailored to the needs of the group/ setting.	• Attendance at group in-venue sessions (Register) • Attendance at Individual sessions (Register) • Facilitator – Group meeting checklist • Facilitator – Individual session checklist • Researcher - Group meeting checklist

Adapted from Bellg et al. (2004) [8]

### Data collection methods

The design of the fidelity framework (see Table 1) and the accompanying data collection instruments were adapted from the experiences of a previous trial of a complex psychosocial intervention conducted by the authors [27]. Throughout, the value of obtaining self-report and multiple perspectives, including the views of those both delivering and receiving the intervention training was prioritised. Consequently, in addition to the researcher observations, we obtained the perspectives of interventionists. Data collection completed by the interventionists also acted as a training tool to reinforce intervention delivery as intended and reduce facilitator drift [8]. However, to encourage completion rates as well as reduce the burden upon interventionists, acceptability and usability were prioritised when designing these data collection tools [17].

Study participants were not asked for their views as part of the fidelity assessment. This was because it was considered too burdensome to ask individuals to engage in activities beyond those already being requested of them, which included completing a number of questionnaires at multiple time points for all and additional qualitative interviews for some.

A range of assessment methods were used to reflect both researcher and interventionists’ perspectives of intervention delivery [8]. Data were collected at multiple time points [30, 31] to enable findings to be compared for similarity or differences over time. Methods used included researcher completion of itemised checklists of non-participatory (unobtrusive) observations [32] of training and of a purposive sample of in-venue group meetings.

Observation is an established research method, enabling understanding of complex relationships and lived reality through observable phenomena and behaviour in context [33]. For this fidelity assessment observations were conducted to enable the researcher to have the same experience as participants whilst remaining detached from the group. Maintaining detachment during an observation can be challenging when present in the same space as participants. A level of engagement is sometimes inevitable for example when making introductions and explaining observer presence or putting participants at ease. In-person observations were selected over video recorded observations in response to learning from previous studies where the technology was found to be challenging for those involved [12].

Researchers also kept comprehensive observation field notes to support and evidence scoring decisions. Attendance registers for both group and individual sessions were analysed as an objective measure of engagement with the intervention [7] and interventionists self-report itemised checklists for the two-day training, in-venue group meetings, individual sessions and supervision were completed by interventionists at the fidelity sites to cross-validate with researcher observations.

All methods were performed in accordance with the relevant guidelines and regulations [34, 35].

### Data collection tools

Assessment tools were developed by the fidelity lead and members of the Trial Management Group (TMG) including the authors of the manualised intervention. Materials used during facilitator training, the manualised intervention and associated resources, and trial protocols all informed the content of these tools. They were all designed to interrogate:

- evidence of learning and skills acquisition
- receipt and enactment of core skills
- anticipated observed behaviours e.g. role-play (for training fidelity) and contributions to the group (for group intervention fidelity).

Since these were purposely designed tools to measure fidelity of the Journeying through Dementia intervention, they were deemed to be sufficiently sensitive to the complexity of the components and contextual variables specific to this intervention [4]. Two researchers, one who managed the feasibility study, scored pre-defined criteria using itemised checklists to rate the core components of the training or intervention [19]. The draft assessment tools were piloted and refined using observations of the first training session and the first group in-venue session at two of the fidelity sites. This process was conducted to identify items that could not be reliably scored by the researchers, improve consistency of scoring and make revisions before further application [36].

Details of the tools are listed below and copies of the final checklists and registers are provided as supplementary document 2.

#### Training observation checklists

To evaluate interventionist training, observation checklists were completed concurrently by two researchers during delivery. A simplified version of the checklist was also completed by the interventionists immediately following training to record what they understood that they had received during the two successive days. Out of a total of six two-day training sessions offered to the interventionists from the four sites, the first three were observed and scored using an itemised checklist by the fidelity lead (KS) and a second researcher (SM or JBD).

#### Supervision registers and checklists

Data reported for fidelity to the supervision protocol here are those completed for two intervention programs at three of the four fidelity sites and three at the fourth. Completion was dependent on how many groups each site delivered. All supervisors were asked to complete a register to record facilitator attendance and the format of every session they conducted (expected to be weekly throughout the entire intervention delivery period). Both facilitators and supervisors completed an itemised fidelity checklist derived from the supervision protocol to detail their views of delivery/receipt of supervision. This was requested at the end of the first, fifth and twelfth weeks of supervision for all interventions delivered.

#### Attendance registers – group meetings and individual sessions

Attendance registers maintained by the facilitators during delivery of all group (including out of venue sessions) and individual sessions recorded compliance with attendance including the offer and acceptance or alternatively decline of sessions by participants.

#### In-venue group meeting observation checklists

To assess fidelity of the in-venue group meetings, the observation checklists were completed by the researchers during delivery of two sessions at each site. Comprehensive notes were also taken to evidence researcher scoring decisions. A simplified version of the checklist was also completed by the facilitators immediately after the group finished. Observations of eight in-venue meetings (one group per site, two meetings per group at approximately week three and week eight of delivery) were conducted in total. Observing two meetings per group also enabled us to examine any learning effects and identify potential facilitator drift as intervention delivery progressed [37].

#### Individual session checklist

Direct observations or recording of individual sessions were considered too intrusive, particularly as these sessions were required to be in the participant’s home or local community [12]. Facilitators were asked to complete a summary fidelity checklist evaluating their experience of delivering these sessions as intended. It was requested that these be completed immediately after each session.

#### Out of venue activities

Out of venue activities were not observed or recorded as there were significant ethical and practical considerations; for example, when interacting in the community with people who were not part of the group or the trial.

## Methods of data analysis

### Time between training and delivery

Time lapse between the dates training was received and when intervention delivery commenced were recorded by the research team. Dates were then extracted from the trial database.

### Completion rates

Checklist and register completion rates were analysed to identify adherence to intervention as intended.

### Descriptive analysis of observational data

Frequency scores from the observation checklists for training and in-venue group sessions were compared to identify researcher rates of agreement for criteria achievement. Where there was disagreement, free text notes taken during observations were referred to reach agreement on the criterion score. The categories for scoring were ‘0’ never observed, ‘1’ sometimes observed and ‘2’ observed most of the time. Several criteria within the checklists were rated on a binary ‘Yes’ or ‘No’ scale for presence or absence in this instance for the purpose of analysis if a criterion was rated ‘Yes’ this was converted to ‘2’ and ‘No’ converted to ‘0’. All interventionist completed checklists were rated binary ‘Yes’ or ‘No’ for ease of completion. Fidelity scores were calculated based on percentage agreement on the final score (after moderation) obtained by each researcher as follows:

- 0–60% Unsatisfactory
- 61–70% Satisfactory
- 71–80% Good
- 81–90% Very good
- 91–100% Excellent

The percentage fidelity score obtained by the researchers on the observation checklists was then compared to that of the interventionists on the associated self-report checklist to look for convergence or divergence.

### Inter-rater reliability

Inter-rater reliability assessed the extent to which the two researchers attributed the same score to the same checklist item during session observations only. A weighted kappa using a predefined table of weights was applied to provide estimates of the degree of agreement between the two researchers. As per Cohens Kappa [38] values for inter-rater agreement are as follows:

- ≤ 0 indicating no agreement
- 0.01–0.20 none to slight
- 0.21–0.40 fair
- 0.41–0.60 moderate
- 0.61–0.80 as substantial
- 0.81–1.00 almost perfect agreement

## Results

### Training fidelity

#### Interventionist training

The first three out of six two-day training sessions were observed. These sessions were attended by a range of sites taking part in the trial in addition to the four fidelity sites as shown in Table 2.

**Table 2:** Two day training delivery and attendance

Training session	Date of training	Fidelity site(s) attended	Date of first group meeting	Trainers	Overall numbers of attendees by role			Research observers
					Facilitators	Supervisors	Total per day	
1	17 Jan 17	Site 4	06 Apr 17	CC/SB	10	1	11	KS/SM
	19 Jan 17			CC/SB	10	1	11	KS/SM
2	23 Jan 17	Site 1 Site 2 Site 3	24 May 17 24 Apr 17 06 Jun 17	CC/SB	21	6	27	KS/SM
	24 Jan 17^a^			CC/SB	21	1	22	KS/SM
3	22 Aug 17	None		CC/GM^b^	7	1	8	KS/JBD^c^
	23 Aug 17			CC	7	1	8	KS/JBD

^a^ Day 2 focusses on planning and delivering a session, due to large numbers of attendees and available time, the supervisors and research assistants were asked not to attend Day 2 on this session

^b^ Due to the timing of this session and availability of the trainers a third trainer was recruited

^c^ Due to staff changes a third researcher undertook scoring

Overall fidelity to the training was excellent in all observed three training sessions as scored by the researchers averaging 95% achievement (range 91–97), see Table 3. Facilitator and supervisor (trainee) scores averaged 94% achievement (range 88–97).

**Table 3:** Coder training overall fidelity score agreement

Training session	Coder 1	Coder 2	Agreed score
17-19th January 2017	75/78	76/78	75/78 (96)
23-24th January 2017	70/78	71/78	71/78 (91)
22-23rd August 2017	76/78	76/78	76/78 (97)

As Table 3 illustrates, the lowest fidelity score was obtained for the second of the three observed two-day training (23rd–24th Jan 2017). This could be explained by the necessary delivery modifications to accommodate the numbers attending, which had exceeded what was planned (see Table 2). These modifications included reducing the time spent on a topic/activity or excluding it altogether as well as limiting opportunities for practical activities/exploration. There was agreement between the researchers and trainees on the top three items that were not delivered during this modified session which were:

- Item 7 ‘Did the trainer discuss the supporter attended sessions and relationship dynamics’ (8 out of 18 trainees)
- Item 11 ‘Were you able to reflect on and share your own facilitation style and skills’ (8 out of 18 trainees).
- Item 17 ‘Did you discuss the value and principles of supervision’ (12 out of 18 trainees). This rating related predominantly to the second training session and was one of the topics affected by the modifications made to day-two of the training.

#### Implementation of training

Analysis of trial records found that on average, the time from training to delivery of a first intervention group at the four sites was 106 days (range 79–134).

### Supervision fidelity

At the fidelity sites, there were no changes to facilitators and supervisors during the assessment period. Supervision registers were completed and returned by 11 facilitators and five supervisors (one site had two supervisors who shared supervision responsibilities). Out of the 111 opportunities for supervision identified and recorded by the four sites 105 were recorded as having been completed. Five supervision sessions were not achieved due to annual or sick leave and one was cancelled by the supervisor. On three occasions, once at each of three sites, supervisors held an additional supervision session prior to commencement of a new intervention programme. Supervision with more than one facilitator, referred to as joint supervision in the supervision protocol, was held on 59 occasions and individual supervision on 42. Four sessions were recorded as being delivered as a combination a joint and individual session within the allocated time. Delivery format included 97 face-to-face sessions and eight using remote methods (telephone or Skype). The average time was 61 min (range 30–125 min) for joint supervision and 51 min (range 25–70 min) for individual supervision. Only one out of the four fidelity sites met the requirements for provision of a minimum of four individual supervision sessions as identified in the protocol. A further two sites did achieve stated requirements during delivery of their second intervention programme after receiving feedback and encouragement from the clinical experts on the trial team.

Supervisors rated their fidelity to the supervision protocol as being very good on all three observation checklists; averaging 82% achievement (range 77–86) (see Table 4). Supervisors recorded that they had delivered most components of supervision during each session. The component that was most frequently not fulfilled was ‘Did you use a reflective diary as part of the supervision session?’ which was optional for facilitators to complete.

**Table 4:** Supervisor completed fidelity scores for individual sessions

Week	Item completion rate/max score (%)	Agreement on completed scores (%)
**1**	81/90 (90)	70/81 (86)
**6**	80/90 (89)	67/80 (84)
**12**	73/80 (91)	56/73 (77)

### Intervention fidelity

#### Attendance registers

Participant attendance was good with 25 out of 35 (71%) participants receiving the therapeutic dose of 10 out of 16 sessions (12 weekly meetings and four individual sessions). Five (14%) attended all 16 sessions.

Group session attendance was good. Of the total 331 available sessions to participants 264 (80%) were attended.

All 35 participants took part in the first individual session. Of those 35, eight participants then withdrew from the intervention between the first and second individual session and one before the third individual session. Of the remaining 26 participants, 25 took part in all four sessions.

#### Group meeting checklist fidelity

Observed fidelity to the group aspect of intervention was very good with researchers reaching between 88 and 95% agreement on observed items delivered (see Table 5). For all eight observed sessions Cohens kappa score of 0.68 demonstrated substantial inter-rater reliability 95% CI (0.58–0.78) between the two researchers.

**Table 5:** Fidelity and Kappa scores by sites

	Researcher 1 score^a^	Researcher 2 score^a^	Agreed Researcher score	%	Kappa score	***P*** value	CI
Site 1	127	124	127/144	88	0.77	p = < 0.05	0.43–0.84
Site 2	137	136	137/144	95	0.64	p = < 0.05	0.39–0.78
Site 3	123	122	123/144	85	0.66	p = < 0.05	0.45–0.86
Site 4	128	132	130/144	90	0.59	p = < 0.05	0.57–0.96

^a^The score is a combined total per researcher for the two in-venue group meetings observed by site

The in-venue group meeting checklists for facilitators were all completed as requested (100%). The recorded ratings reflected those of the observing researchers with fidelity across all eight groups averaging 93% (84–100).

#### Individual session checklist fidelity

Individual session checklists for a total of 20 participants (out of a possible 35) were completed by the facilitators as part of intervention delivery. Seven of the 20 participants had incomplete records that could not be accounted for by intervention withdrawal. An average of 77% achievement (range 22–100) was found for items delivered during each session. The two lowest scored items for delivery were item 5 with 40% achievement ‘Did you help the participant set any goals’ and item 8 with only 22% achievement ‘Did you enable the participant to rehearse skills learned in their everyday life’.

## Discussion

The aim of this embedded fidelity assessment was to determine how well the Journeying through Dementia intervention was delivered according to the trial protocol and intervention manual. A second aim was to further examine what methodologies can be practically, ethically and reliably employed for assessment of fidelity during pragmatic trials of complex interventions.

The assessment tools (checklists and registers) derived from the fidelity framework were found to be fit for purpose suggested by good completion rates and inter-rater reliability, and we were able to demonstrate compliance with the intervention and fidelity to delivery of the in-venue group component of the intervention. The caveat here was that we were not able to observe all core components of the intervention due to the inability to be able to capture the delivery of out of venue and individual sessions. Interwoven with this were ethical considerations such as participant rights to confidentiality and the intrusive and impractical nature of observation of these sessions [12]. Indeed, the findings from the qualitative sub study [39] indicated that the components that we were not able to assess were the more challenging aspects for facilitators to deliver and where participant behaviour change was critical for success. Examples included enabling participants to practice learning in the community with the support of others and being assisted to identify and work towards individual goals. The most appropriate methods for monitoring fidelity in psychosocial community-based complex interventions are therefore yet to be identified.

### Methodological challenges

Understanding social constructs and interpreting observed behaviours are influenced by the subjective interpretations of researchers [40]. The inherent challenges of measuring the subjective nature of observed outcomes of complex interventions such as those being promoted through Journeying through Dementia as well as the observation method itself means by design, any bespoke instruments will lack psychometric properties. Researchers have little choice but to use bespoke assessment tools to evaluate behavioural complex interventions and such tools need to be designed underpinned by intervention theory and content [4].

Certain behaviours and criteria detailed on the observation measure were more concrete to understand and therefore observe and score. One example was concerned with the practicalities of delivery, and whether two facilitators were present as required by the protocol. In comparison, other criteria were more subjective such as relying on the researcher observing and recognising specific behaviours, for example evidence of facilitators enabling participants. This required the researchers who were scoring to fully understand how behaviours manifest, and use their judgement when interpreting what they observed [41]. A significant amount of prior work was therefore needed by those who were going to undertake the observations to agree how to identify and rate the criteria on the observation checklist [42]. Piloting was essential and it quickly established that several criteria were unlikely to be observed during the in-venue group aspect of the intervention over a limited number of sessions, for example practicing learning in the community. It is essential that those who are to observe and score intervention fidelity are involved in identifying how criteria might manifest and how to score criteria. Therefore the recommendation is for observers to take comprehensive notes in addition to scoring the checklist to use as evidence to explain scoring decisions.

Evaluation of this intervention required a comprehensive and detailed understanding of the manualised programme and training package. The primary trainer was the author of the manual and some members of the research team including the Chief Investigator and Fidelity Lead had gained extensive experience of the manual during its use in the prior feasibility study [24]. This knowledge and experience aided the development of the conceptual framework for fidelity assessment and creation of assessment instruments. One of the observing researchers had detailed knowledge of the intervention from working on the feasibility study. Whether someone who is not fully immersed, or supported by an experienced person, in this intervention can understand and therefore score the nuances is debatable and we therefore posit that inclusion of researchers with experience of the intervention for fidelity assessment of complex interventions is preferable.

The tools and measures used for this fidelity assessment were based in the underpinning methodology and ethos of the intervention, yet the question of robustness remains [43, 44]. To increase credibility we incorporated data from multiple sources including from the researchers and from the perspective of interventionists via self-report [30, 31]. However, the influence of observer presence and interactions as well as the inclusion of the interventionists’ self-report may have resulted in the Hawthorne effect and therefore reporting bias, with individuals potentially trying harder to achieve optimum scores on the fidelity checklists. As observers only attended two meetings at each site the fidelity researchers’ presence was evident to both the facilitators and participants and had to be explained. However, our findings suggest that the similarity in scores obtained from the researchers and interventionists indicated that potential for bias was limited. In addition conducting observations over time [33] and use of multiple perspectives helped reduce these potential bias [10]. We are however unable to comment on how comparable the remaining eight sites were in their intervention delivery to the four fidelity sites.

The research developed and piloted bespoke itemised checklists for observations including agreement of scoring guidelines. Facilitators however, although given simplified versions of the checklist with binary scoring (Yes/No) for usability, were not asked to score observations but to indicate whether they recalled a specified item being completed or achieved. Therefore, inconsistencies may have occurred based on memory recall for the facilitators. However, as our findings found similarity in scores obtained from the observing researchers and from facilitator’ self-report, this potential bias was limited. Encouraging and implementing simple and effective protocols for timely completion of self-report checklists could assist with completion rates as well as quality of data.

Timing of training in trials of group interventions is a well-known challenge [45]. The time lag between training and delivery of the intervention could have impacted upon fidelity but we mitigated against this by site supervisors being place and supervision of the site supervisors by the research team.

Lastly the assessment process was not applied to the individual sessions or out of venue activities due to several ethical considerations. These included the need to maintain participant confidentiality during individual sessions. It is essential this component of the intervention is seen as a safe space for the person to speak freely if they are to maximise participation in the intervention. And secondly confidentiality and consent issues when interacting with people who were not part of the trial during community based out of venue activities. We therefore relied on facilitator self-reports for these components of the intervention. We did consider asking participants themselves to provide self-report on these components but felt that due to other trial requirements this was deemed too burdensome. Obtaining participants contemporary views of the intervention would however provide nuanced data to better understand the intervention in action. If we are to evidence fidelity to delivery as intended as well as in relation to intervention effectiveness then all components of complex interventions need to be included in assessments.

## Conclusion

We have conducted a fidelity evaluation of a complex psychosocial intervention, demonstrating its fitness for assessing several core components specific to the intervention. In addition, we have demonstrated that non-participatory observations in the community are possible when carried out in a regular venue. This approach can be used as a model for development of fidelity assessment for community based complex interventions. However, we were not able to observe all core components of the intervention due to the inability to be able to either observe or record all aspects. Therefore, questions remain about how to best observe and assess fidelity in community based complex psychosocial interventions where methodological and ethical issues prevent use of established assessment methods.

## Supplementary Information

**Additional file 1.**

## Data Availability

The datasets generated and analysed for this study will be available upon request from the corresponding author.
