# Supplementary material for: Assessing fidelity of a community based psychosocial intervention for people with mild dementia within a large randomised controlled trial
Source: BMC Geriatr. 2021 Feb 11;21:119. doi: 10.1186/s12877-021-02070-8 (PMC7879661; doi:10.1186/s12877-021-02070-8)
Supplement: Supplementary file 1 — Additional file 1. [file 12877_2021_2070_MOESM1_ESM.pdf]

SUPPLEMENTARY DOCUMENTS

Assessing fidelity of a community based psychosocial intervention for people with mild dementia within a large randomised controlled trial

Kirsty Sprange[1*], Jules Beresford-Dent[2], Gail Mountain[3], Claire Craig[4], Clare Mason[3], Katherine Berry[5], Jessica Wright[6], Shazmin Majid[7], Ben Thomas[6], Cindy L Cooper[6]

[1] Kirsty Sprange*, Nottingham Clinical Trials Unit, Faculty of Medicine, University of Nottingham, Nottingham NG7 2RD

[2] Jules Beresford Dent, University of Bradford, Bradford West Yorkshire BD7 1DP, UK

[3] Gail Mountain, University of Bradford, Bradford West Yorkshire BD7 1DP

[4] Claire Craig, Sheffield Hallam University, City Campus, Sheffield, S1 1WB

[3] Clare Mason, University of Bradford, Bradford West Yorkshire BD7 1DP

[5] Katherine Berry, Manchester Academic Health Science Centre, The University of Manchester, Oxford Rd, Manchester, M13 9PL

[6] Jessica Wright, Sheffield Clinical Trials Research Unit, School of Health and Related Research, University of Sheffield, Sheffield, S1 4DP

[7] Shazmin Majid, Institute of Mental Health, Jubilee Campus, University of Nottingham Innovation Park, Triumph Road, Nottingham NG7 2TU

[6] Ben Thomas, Sheffield Clinical Trials Research Unit, School of Health and Related Research, University of Sheffield, Sheffield, S1 4DP

[6] Cindy Cooper, Sheffield Clinical Trials Research Unit, School of Health and Related Research, University of Sheffield, Sheffield, S1 4DP

Supplementary Document 1: Journeying through Dementia Supervision Protocol

Journeying through Dementia
Supervision Protocol

This protocol provides a framework for supervision of facilitators delivering the Journeying through Dementia programme.

1. **Overall aims of supervision**
   - To reinforce the importance and value of the facilitator role.
   - To guide the facilitator to deliver the intervention effectively to meet the needs of the participants and in accord with the Journeying through Dementia Programme as in the manual provided.
   - To provide the facilitator with an opportunity to discuss the group and 1:1 sessions including any challenges.
   - To assist the facilitator to reflect upon their practice, identify their own solutions to emergent issues and continually improve methods of intervention delivery over time.
   - To identify any further learning and development needs in response to progress over time.

2. **General principles**

2.1. The designated supervisor will attend relevant training to support the facilitators employed to deliver the Journeying through Dementia programme.

2.2. Supervisors will take time to become familiar with the manualised intervention and associated publications and understand the role of the facilitator within the programme.

2.3. Supervisors will arrange regular one-to-one supervision meetings with designated facilitators and aim to continue supervising the same individual(s) during intervention delivery.

2.4. One-to-one supervision meetings will be arranged at a mutually convenient time and place. Meetings are expected to take place weekly of which at least half should be conducted face-to-face. At least four sessions should be conducted on an individual basis with optional joint supervision for the remaining sessions as agreed. Distance supervision can include phone and skype calls.

2.5. Supervisors will complete a Contact Log for each weekly session.

2.6. The specific methods and content of supervision may vary between individuals and will be assessed by the supervisor. Overall the process will encourage self-assessment, reflective and analytical skills. Depending upon the individual these may include;

I. Responding to the complexity of facilitator needs
II. Determining facilitator skills including strengths and skill gaps
III. Providing feedback on progress with intervention delivery
IV. Providing guidance to try and ensure that the requirements set out by the programme are met

2.7. Facilitators may maintain a reflective diary to record the progress of weekly group meetings and 1:1 sessions and reflect upon their own progress. The contents of this diary can be discussed during supervision sessions but only if the facilitator decides that this should happen.

3. Content

3.1. Supervision sessions may cover the following issues (depending upon the individual needs of a facilitator):

I. Pre planning of groups
II. How sessions progressed
III. Ideas of how it might have been improved
IV. How to tailor the groups to the needs of the participants whilst adhering to the Journeying through Dementia programme and manual
V. Any specific issues for group members and how they might be taken forward
VI. Progress with individual sessions - ideas of how they might be improved/ lead to achievement of better outcomes
VII. Facilitator's perceptions of own abilities / need for support to undertake role
VIII. Provision of assistance with improving performance

4. Supervisor/Supervisee Contract (1)

4.1. To work together to facilitate an in depth reflection on issues relating to practice.

4.2. To meet on a weekly basis for 1hr with each individual facilitator.

4.3. To protect and value the time and space for supervision.

4.4. To respect each other's views and be open to feedback.

4.5. To pre-arrange each session at the end of the previous session, or establish a regular date and time. If a session is cancelled to try and rearrange if time allows.

4.6. Note taking during sessions will be the responsibility of the supervisor and the facilitator. It is essential that this information is kept confidential and participants are not identifiable. The only exceptions will be if issues are discussed which compromise any Professional Code of Conduct/Vulnerable adult issues.

4.7. The supervisor will maintain an aide memoir of supervision after each session.

4.8. Sessions will be confidential but not secret, if anything is disclosed that is pertinent for the research team to know in terms of facilitator or participant health and safety you will have a duty of care to report this to the study Chief Investigator and the responsible clinician.

4.9. The supervisor will inform the line manager of any concerns about the performance of the facilitator role and/or their delivery of the intervention, as soon as possible.

5. Supervision logs

5.1 The supervisor will complete the JtD Supervisor Contact Log on a weekly basis and the JtD Supervision Booklet at weeks 1, 5 and 12 of supervision.

6. Line management

6.1. Will be provided by the employer.

Supplementary Document 2: Journeying through Dementia Fidelity Data Collection Tools

1. Journeying through Dementia: Training Delivery checklist for Researchers

Scale: 0=**None** of the time; 1= **Some** of the time; 2=**Most** of the time; 3= **All** of the time OR ✓equals a score of 3, a X equals a score of 0

**TRAINER**				
**Domain**	**Criteria**	**Scale**	**Independent Score**	**Agreed Score**
**Trainer**	1. The trainer is the same for all training	✓or X		
	2. The trainer is an author or skilled in the JtD intervention	✓or X		
	3. Did the trainer model a range of group work skills:<br>• teaching<br>• peer sharing<br>• interactive activities<br>• active experimentation (Testing things out)	 ✓or X<br>✓or X<br>✓or X<br>✓or X		
**Materials**	4. PowerPoint presentations and training materials directly relate to the goals of the training	0 - 3		
	5. Provides handouts and materials to all trainees	✓or X		
**Tailoring**	6. Does the trainer elicit information about skills levels/previous experience	0 - 3		
	7. Training is tailored in response to the observed needs of the trainees, and feedback from the trainees during the sessions	0 - 3		
**Content**	8. Discuss dementia and its impact	✓or X		
	9. Discuss the evidence for JtD	✓or X		
	10. The goals of the training are covered	✓or X		
	11. Examples of topics are covered from the manual	✓or X		
	12. Mandatory themes are covered:<br>• A celebration of achievements<br>• Activity and health<br>• Endings	 ✓or X<br>✓or X<br>✓or X		
	13. Individual sessions<br>• Purpose<br>• Goal setting<br>• Participant relationship with supporter<br>• How the participant & supporter work together on the programme	 ✓or X<br>✓or X<br>✓or X<br>✓or X		
	14. Discuss supporter attended sessions and relationship dynamics	✓or X		

TRAINEES				
**Domain**	**Criteria**	**Scale**	**Independent Score**	**Agreed Score**
**Understanding**	15. Identify and understand the ideas that are key to JtD inc:<br>• Enabling group members to do things for themselves<br>• Transferring skills from the safety of the group to their wider lives	 0 - 3<br>0 - 3		
	16. Discuss the content of the manual and how to apply it	0 - 3		
**Self - awareness**	17. Share their prior expertise, hopes and expectations of the training	0 - 3		
	18. Share their own perceptions of dementia and how this may impact on delivering the intervention	0 - 3		
	19. Reflect on and share their own facilitation style and skills	0 - 3		
**Skill acquisition**	20. Use the JtD manual to practice planning sessions	0 - 3		
	21. Identify how to locate and access community resources	0 - 3		
	22. Discuss strategies for peer support	0 - 3		
	23. Take part in and contribute to potential scenarios	0 - 3		
**Group work**	24. Understand and demonstrate a range of group work skills (didactic teaching, peer sharing, interactive activities, active experimentation etc)	0 - 3		
	25. Understand and demonstrate how to manage group dynamics	0 - 3		
	26. Demonstrate how to tailor the intervention to the needs and choices of group members	0 - 3		
**Individual sessions**	27. Explore the value of 1:1 sessions and potential activities to use in these	0 - 3		
**Group outings**	28. Explore the value of outings and how to help group members to plan them	0 - 3		
**Reflection & evaluation**	29. Explore reflective practice through feedback within the training and the group	0 - 3		
**Supervision**	30. Discuss the value and principles of supervision	0 - 3		

2. Journeying through Dementia: Training Delivery checklist for trainees

Questions	
1. Did the trainer use a range of group work skills, for example teaching, peer sharing, interactive activities?	Yes ☐ No ☐
2. Were presentations and training materials related to the objectives of the training?	Yes ☐ No ☐
3. Did you feel the training was tailored to your understanding and/or experience?	Yes ☐ No ☐
4. Did the trainer(s) respond to feedback from the trainees during the sessions?	Yes ☐ No ☐
5. Were examples of topics covered from the manual?	Yes ☐ No ☐
6. Did the trainer discuss the individual sessions including their purpose, goal setting and relationships (between participant and carer)?	Yes ☐ No ☐
7. Did the trainer discuss the supporter attended sessions and relationship dynamics?	Yes ☐ No ☐
8. Were you able to discuss the content of the manual and how to apply it?	Yes ☐ No ☐
9. Were you able to share your expertise with the group and your expectations of the training?	Yes ☐ No ☐
10. Were you able to share your own perceptions of dementia and how this may impact on delivering the intervention?	Yes ☐ No ☐
11. Were you able to reflect on and share their own facilitation style and skills?	Yes ☐ No ☐
12. Did you use the JtD manual to practice planning sessions?	Yes ☐ No ☐
13. Did you discuss strategies for peer support?	Yes ☐ No ☐
14. Did you discuss how to manage group dynamics?	Yes ☐ No ☐
15. Did you discuss how to tailor the intervention to the needs and choices of group members?	Yes ☐ No ☐
16. Did you explore the value of 1:1 sessions and potential activities to use in these?	Yes ☐ No ☐
17. Did you discuss the value and principles of supervision?	Yes ☐ No ☐

3. Supervision Register

**Week Number**	**Name(s) of facilitator(s) attending:** (please list)	**If someone did not attend please state name and reason why:** (e.g. unwell, too busy)	**Type of supervision** (tick one box)	**Mode of delivery** (tick one box)	**Length of time (e.g. mins/hrs)**	**Any further action required by the supervisor**
Pre-Group*			Individual ☐<br>Joint/group ☐	Face-to-face ☐<br>Tel/skype ☐		
Week 1			Individual ☐<br>Joint/group ☐	Face-to-face ☐<br>Tel/skype ☐		Complete checklist for 'Week 1' (page 4)
Week 2			Individual ☐<br>Joint/group ☐	Face-to-face ☐<br>Tel/skype ☐		
Week 3			Individual ☐<br>Joint/group ☐	Face-to-face ☐<br>Tel/skype ☐		
Week 4			Individual ☐<br>Joint/group ☐	Face-to-face ☐<br>Tel/skype ☐		
Week 5			Individual ☐<br>Joint/group ☐	Face-to-face ☐<br>Tel/skype ☐		Complete checklist for 'Week 5' (page 5)
Week 6			Individual ☐<br>Joint/group ☐	Face-to-face ☐<br>Tel/skype ☐		
Week 7			Individual ☐<br>Joint/group ☐	Face-to-face ☐<br>Tel/skype ☐		
Week 8			Individual ☐<br>Joint/group ☐	Face-to-face ☐<br>Tel/skype ☐		
Week 9			Individual ☐<br>Joint/group ☐	Face-to-face ☐<br>Tel/skype ☐		
Week 10			Individual ☐<br>Joint/group ☐	Face-to-face ☐<br>Tel/skype ☐		
Week 11			Individual ☐<br>Joint/group ☐	Face-to-face ☐<br>Tel/skype ☐		
Week 12			Individual ☐<br>Joint/group ☐	Face-to-face ☐<br>Tel/skype ☐		Complete checklist for 'Week 12' (page 6)

4. Week 1 Supervision Checklist

Question	Facilitator 1	Facilitator 2<br>Not applicable ☐
1. Did you read the Supervision protocol prior to arranging and/or conducting supervision?	Yes ☐<br>No ☐	Yes ☐<br>No ☐
2. Did you use or refer to the JtD manual during the supervision session?	Yes ☐<br>No ☐	Yes ☐<br>No ☐
3. Did you refer to any aspect of the 2 day training during the supervision session?	Yes ☐<br>No ☐	Yes ☐<br>No ☐
4. Did you use a reflective diary as part of the supervision session?	Yes ☐<br>No ☐	Yes ☐<br>No ☐
5. Did you discuss planning of group sessions?	Yes ☐<br>No ☐	Yes ☐<br>No ☐
6. Did you discuss group dynamics?	Yes ☐<br>No ☐	Yes ☐<br>No ☐
7. Did you discuss how to tailor the programme for the group?	Yes ☐<br>No ☐	Yes ☐<br>No ☐
8. Did you discuss the 1-1 sessions?	Yes ☐<br>No ☐	Yes ☐<br>No ☐
9. Did you offer guidance/problem solving?	Yes ☐<br>No ☐	Yes ☐<br>No ☐

Week 5 Supervision Checklist

Question	Facilitator 1	Facilitator 2<br>Not applicable ☐
1. Did you use or refer to the JtD manual during the supervision session?	Yes ☐<br>No ☐	Yes ☐<br>No ☐
2. Did you refer to any aspect of the 2 day training during the supervision session?	Yes ☐<br>No ☐	Yes ☐<br>No ☐
3. Did you use a reflective diary as part of the supervision session?	Yes ☐<br>No ☐	Yes ☐<br>No ☐
4. Did you discuss planning of group sessions?	Yes ☐<br>No ☐	Yes ☐<br>No ☐
5. Did you discuss group dynamics?	Yes ☐<br>No ☐	Yes ☐<br>No ☐
6. Did you discuss how to tailor the programme for the group?	Yes ☐<br>No ☐	Yes ☐<br>No ☐
7. Did you discuss the 1-1 sessions?	Yes ☐<br>No ☐	Yes ☐<br>No ☐
8. Did you offer guidance/problem solving?	Yes ☐<br>No ☐	Yes ☐<br>No ☐
9. Did you discuss enactment of activities (outings) in the community	Yes ☐<br>No ☐	Yes ☐<br>No ☐

Week 12 Supervision Checklist

Question	Facilitator 1	Facilitator 2<br>Not applicable ☐
1. Did you use or refer to the JtD manual during the supervision session?	Yes ☐<br>No ☐	Yes ☐<br>No ☐
2. Did you refer to any aspect of the 2 day training during the supervision session?	Yes ☐<br>No ☐	Yes ☐<br>No ☐
3. Did you use a reflective diary as part of the supervision session?	Yes ☐<br>No ☐	Yes ☐<br>No ☐
4. Did you discuss planning of group sessions?	Yes ☐<br>No ☐	Yes ☐<br>No ☐
5. Did you discuss group dynamics?	Yes ☐<br>No ☐	Yes ☐<br>No ☐
6. Did you discuss how to tailor the programme for the group?	Yes ☐<br>No ☐	Yes ☐<br>No ☐
7. Did you discuss the 1-1 sessions?	Yes ☐<br>No ☐	Yes ☐<br>No ☐
8. Did you offer guidance/problem solving?	Yes ☐<br>No ☐	Yes ☐<br>No ☐

5. Journeying through Dementia: Group Meeting Delivery checklist for Researchers

Scale: 0=**None** of the time; 1= **Some** of the time; 2=**Most** of the time ✓equals a score of 3, a **X** equals a score of 0						
**FACILITATOR**						
**Domain**	**Criteria**	**Scale**	**Coder 1 Score**	**Coder 2 Score**	**Agreed Score**	**Comment/ Evidence**
Resources	1. Two facilitators deliver the group	✓or X				
	2. Uses range of teaching techniques and/or materials e.g. flipcharts as aide memoirs	✓or X				
	3. Utilises the manual (menu of topics) and/or associated activities	✓or X				
	4. Discusses use of resources e.g. community, technology, the group itself	✓or X				
Personal qualities	5. Shows warmth and empathy	✓or X				
	6. Listens actively and respectfully the group	✓or X				
Enabling	7. Builds on the assets, skills and abilities that each person brings	0 - 2				
	8. Encourages the group to make choices and supports these	0 - 2				
	9. Grading and layering of activities to extend skills and build confidence	0 - 2				
Group work skills	10. Facilitates communication and interaction	0 - 2				
	11. Ensuring participants are not overwhelmed e.g with too much information	0 - 2				
	12. Draws in quiet members of the group	0 - 2				
	13. Supports the group managing use of space and time effectively	0 - 2				
	14. Supports the group managing noise levels and tempo of the sessions to ensure discussions are not rushed allowing people to contribute	0 - 2				
	15. Facilitates the group exploring group dynamics, or conflict/resolution	0 - 2				
	16. Builds mutual support and group cohesion	0 - 2				
Goals and needs	17. Balances meeting individual needs and goals and group needs	0 - 2				
	18. Balances challenge in activities with ensuring they are achievable – tailoring the intervention	0 - 2				
Content	19. Focuses on manual topics, or new topics chosen by group members	0 - 2				
	20. Uses activities (manual or from elsewhere), not just discussion	0 - 2				
	21. With members, selects and plans group activities and/or outings that explore and extend the themes in the programme	0 - 2				
	22. Explores techniques to compensate for ways in which dementia impacts on everyday life	0 - 2				

Scale: 0= **None** of the members; 1= **Some** of the members; 2= **Most** of the members						
**GROUP MEMBERS**						
**Domain**	**Criteria**	**Scale**	**Coder 1 Score**	**Coder 2 Score**	**Joint Score**	**Comment**
Understanding	23. Explore importance of the relationship between activity, health and wellbeing	✓or X				
	24. Opportunity to share and discuss feelings about the impact of dementia in context of the topic	0 - 2				
Occupational self-analysis	25. Discuss daily occupations (their significance, impact on health, components) and make choices	0 - 2				
	26. Sharing and reflecting on experiences of participating in activities (Occupational storytelling)	0 - 2				
Goal setting	27. Goals identified within the group and/or within activities	0 - 2				
Mutual support	28. Show mutual appreciation and support	0 - 2				
	29. Achievements are celebrated	0 - 2				
Creativity	30. Practice of self-expression or innovation within the safety of the group	0 - 2				
	31. Practice of problem solving or adaptation within the safety of the group	0 -2				
	32. Appear to be engaging in enjoyable and stimulating activities	0 - 2				
Participants as experts	33. Acknowledge and appreciate their own skills and how they can contribute to the group	0 - 2				
	34. Bring in and share their own skills and knowledge	0 - 2				
	35. Group members in turn take leadership or contribute ideas	0 - 2				
	36. Build skills and/or learn solutions within the group	0 - 2				

6. Journeying through Dementia: Programme Delivery checklist for Facilitators

Questions	
1. Did you use a topic from the manual or cover a new topic during the meeting?	Yes ☐ No ☐
2. Did you explore the importance of the relationship between activity, health and wellbeing in the topic covered during the meeting?	Yes ☐ No ☐
3. Did you discuss how participants could access resources to support the topic e.g. local community, technology, skills within the group?	Yes ☐ No ☐
4. Did you encourage the group to make choices on topics and supports these choices?	Yes ☐ No ☐
5. Were you able to help people communicate effectively in the group?	Yes ☐ No ☐
6. Were you able to facilitate peer exchange and sharing of ideas and experiences within the group?	Yes ☐ No ☐
7. Did you feel you encouraged mutual support in the group and group cohesion e.g. people working together/getting along?	Yes ☐ No ☐
8. Were you able to balance ensuring activities were challenging with ensuring they were achievable for all participants (i.e. tailoring the intervention)?	Yes ☐ No ☐
9. Did you use group activities, for example a quiz, a taster session, and not just discussion to explore the topic?	Yes ☐ No ☐
10. Did you discuss or reflect on any new or existing goals identified by the group?	Yes ☐ No ☐
11. Do you think the participants engaged in enjoyable and stimulating activities?	Yes ☐ No ☐
12. Do you think participants acknowledged and appreciated their own skills and how they contributed to the group?	Yes ☐ No ☐
13. Do you think participants were able to build skills and/or learn solutions within the group?	Yes ☐ No ☐

7. Journeying through Dementia: Individual Session checklist for Facilitators

Questions	
1. Did you explain the importance and purpose of the individual session?	Yes ☐<br>No ☐
2. Did you feel that you have established or built on the participant/facilitator relationship? E.g. mutual regard and trust	Yes ☐<br>No ☐
3. Did the participant have an opportunity to voice any worries, fears or concerns?	Yes ☐<br>No ☐
4. Did you explore any issues or goals of importance to the participant?	Yes ☐<br>No ☐
5. Did you help the participant set any goals?	Yes ☐<br>No ☐
6. Did you talk to the participant about managing change in a positive way?	Yes ☐<br>No ☐
7. Did you enable the participant the opportunity to explore ideas and topics	Yes ☐<br>No ☐
8. Did you enable the participant to rehearse skills learned in their everyday life?	Yes ☐<br>No ☐
9. Did you explore the participant's relationships with family and friends?	Yes ☐<br>No ☐
10. Did you reflect on the group meetings and the participants understanding of what had been covered?	Yes ☐<br>No ☐
